# Reciprocal Expression Patterns of Placental Leucine Aminopeptidase/Insulin-Regulated Aminopeptidase and Vasopressin in the Murine Brain

**DOI:** 10.3389/fmolb.2020.00168

**Published:** 2020-07-24

**Authors:** Yoshikuni Goto, Takahiro J. Nakamura, Kenji Ogawa, Akira Hattori, Masafumi Tsujimoto

**Affiliations:** ^1^Faculty of Pharmaceutical Sciences, Teikyo Heisei University, Nakano, Japan; ^2^Laboratory of Animal Physiology, School of Agriculture, Meiji University, Kawasaki, Japan; ^3^Laboratory of Veterinary Epizootiology, Department of Veterinary Medicine, Nihon University, Fujisawa, Japan; ^4^Department of System Chemotherapy and Molecular Sciences, Graduate School of Pharmaceutical Sciences, Kyoto University, Kyoto, Japan

**Keywords:** placental leucine aminopeptidase, insulin-regulated aminopeptidase, aminopeptidase, vasopressin, circadian rhythm, brain

## Abstract

Placental leucine aminopeptidase/insulin-regulated aminopeptidase (P-LAP/IRAP) regulates vasopressin and oxytocin levels in the brain and peripheral tissues by controlled degradation of these peptides. In this study, we determined the relationship between P-LAP/IRAP and vasopressin levels in subregions of the murine brain. P-LAP/IRAP expression was observed in almost all brain regions. The expression patterns of P-LAP/IRAP and vasopressin indicated that cells expressing one of these protein/peptide were distinct from those expressing the other, although there was significant overlap between the expression regions. In addition, we found reciprocal diurnal rhythm patterns in P-LAP/IRAP and arginine vasopressin (AVP) expression in the hippocampus and pituitary gland. Further, synchronously cultured PC12 cells on treatment with nerve growth factor (NGF) showed circadian expression patterns of P-LAP/IRAP and enzymatic activity during 24 h of incubation. Considering that vasopressin is one of the most efficient peptide substrates of P-LAP/IRAP, these results suggest a possible feedback loop between P-LAP/IRAP and vasopressin expression, that regulates the function of these substrate peptides of the enzyme *via* translocation of P-LAP/IRAP from intracellular vesicles to the plasma membrane in brain cells. These findings provide novel insights into the functions of P-LAP/IRAP in the brain and suggest the involvement of these peptides in modulation of brain AVP functions in hyperosmolality, memory, learning, and circadian rhythm.

## Introduction

Placental leucine aminopeptidase (P-LAP) was first purified from retroplacental serum as a soluble protein (Tsujimoto et al., 1992). Subsequent cloning and sequence analysis of its cDNA revealed that the enzyme is a type II membrane protein localized in intracellular vesicles (Rogi et al., 1996). During pregnancy, P-LAP is synthesized as a vesicular membrane protein in the placenta, cleaved by ADAM12, and then secreted into the maternal serum (Ito et al., 2004). P-LAP activity in the maternal serum remains low in the first trimester, rises progressively during the second and third trimesters, and then declines rapidly after parturition (Yamahara et al., 2000). Therefore, serum P-LAP level has been reported to be a viable predictor of fetal death (Tian et al., 2016). In female Bornean orangutans that gave stillbirths, the P-LAP concentration in urine failed to increase; whereas in those that gave live births, the average P-LAP concentration in urine showed a progressive increase till delivery (Kinoshita et al., 2017). P-LAP is believed to prevent the premature onset of uterine contractions by degrading oxytocin, thereby playing a role in the maintenance of normal pregnancy (Yamahara et al., 2000).

Keller et al. (1995) have reported the cloning of insulin-regulated membrane aminopeptidase (IRAP), which is a rat ortholog of P-LAP and co-localizes with the glucose transporter 4 (GLUT4) in intracellular vesicles. Microinjection of a GST fusion protein containing the cytoplasmic domain of IRAP was shown to cause the translocation of GLUT4-containing vesicles to the plasma membrane in 3T3-L1 adipocytes, suggesting that the *N*-terminal region of IRAP plays a role in the distribution of these vesicles for glucose uptake into the cells (Ross et al., 1996). However, the pathophysiological significance of IRAP (hereafter referred to as P-LAP/IRAP) in diabetes remains elusive (Keller, 2004; Bogan, 2012).

Placental leucine aminopeptidase/insulin-regulated aminopeptidase (P-LAP/IRAP) is a multifunctional enzyme that plays several pathophysiological roles. This enzyme can act as a receptor for angiotensin IV (Ang IV), which facilitates memory retention and retrieval (Albiston et al., 2001). Since Ang IV is an inhibitor of P-LAP/IRAP ligands to the Ang IV receptor are considered to exert memory-enhancing effects by modulating the enzymatic activity of P-LAP/IRAP. Similar to the case of GLUT4-containing vesicles, P-LAP/IRAP is involved in vesicular trafficking of the somatostatin type 2A receptor to the plasma membrane in hippocampal neurons and thus exerts an inhibitory effect on seizure activity (De Bundel et al., 2015). Although P-LAP/IRAP and oxytocin are co-expressed in hypothalamic neuronal cells, these are packaged in separate vesicles (Tobin et al., 2014). Inhibition of P-LAP/IRAP activity in the hypothalamus leads to increased frequency of milk ejection reflexes due to enhancement in oxytocin concentration. These results indicate that P-LAP/IRAP plays important roles in the modulation of several brain functions. This enzyme is also an important player in host defense systems, such as antigen cross-presentation, and Toll-like receptor 9 signaling (Saveanu et al., 2009; Babdor et al., 2017). P-LAP/IRAP trims antigenic peptides presented by MHC class I molecules, and also modulates Toll-like receptor 9 trafficking to lysosomes *via* interaction with FHOD4 (Saveanu et al., 2009; Babdor et al., 2017).

We have earlier reported the expression of P-LAP/IRAP in the brain (Matsumoto et al., 2001). We also identified several neuronal peptide substrates of the enzyme, including dynorphin A, vasopressin, oxytocin, and somatostatin (Tsujimoto et al., 1992; Matsumoto et al., 2000). However, the relationship between the expression levels and activity of P-LAP/IRAP and the expression levels of its substrates in each sub-region of the brain remains unknown. In this study, we examined the roles of this enzyme in the brain. We identified reciprocal rhythmic increases and decreases in P-LAP/IRAP and arginine vasopressin (AVP) levels in several regions of the brain. Vasopressin is known to play multiple roles in distinct regions of the brain, related to hyperosmolality, memory, learning, and circadian rhythm (Johnston, 1985; Alescio-Lautier and Soumireu-Mourat, 1998; Ingram et al., 1998). The functional significance of the present results is discussed in the context of vasopressin being one of the most efficient peptide substrates of P-LAP/IRAP (Tsujimoto et al., 1992; Matsumoto et al., 2000).

## Materials and Methods

### Animals

Male C57BL/6J mice were obtained from the Charles River Laboratories, Japan (Yokohama, Japan). Animal husbandry and all animal experiments were conducted in accordance with the guidelines of the Science Council of Japan and were approved by the Institutional Animal Care and Use Committee of Teikyo-Heisei University. The animals were maintained in a controlled environment (room temperature: 24 ± 1°C; humidity: 50 ± 5 %), with food and water available *ad libitum*, and were housed under a light/dark (LD) cycle of 12 h of light (light intensity: 200–300 lux), and 12 h of darkness until sacrifice.

### Preparation of Brain Extracts

Male mice aged 4–6 months were euthanized at zeitgeber time (ZT) 8–10. ZT is a 24-h normalized notation of the phase in a circadian cycle entrained to the LD condition, with lights on from ZT 0 to ZT 12. The olfactory bulb, pituitary gland, cerebrum, cerebellum, hippocampus, hypothalamus, and thalamus were surgically collected and stored at −80°C for processing. Brain extracts were prepared by homogenizing the whole brain with 0.5% Triton X-100 in 4 mL of cold phosphate-buffered saline (PBS) using a Dounce homogenizer. The homogenate was centrifuged at 15,000 × *g* for 30 min at 4°C, and the supernatant was stored at −80°C until use.

### Immunofluorescence Staining

Whole brains from C57BL/6J mice aged 4–6 months were washed once with cold PBS, fixed by treatment with 4% paraformaldehyde for 24 h at 4°C, and cryoprotected in 30% sucrose in PBS for 48 h. Brain sections were prepared from free-floating coronal brain slices obtained from the middle of the rostrocaudal axis with a CM3050 S cryostat (Leica Biosystems, Richmond Hill, ON, Canada). The sections were washed thrice with PBS containing 0.5% Triton X-100 for 5min each and then blocked with 5% donkey serum (Sigma-Aldrich) for 2 h at room temperature. Next, they were incubated overnight with anti-vasopressin antibody (Catalog No. 20069, ImmunoStar, Hudson, WI, United States) in PBS containing 5% donkey serum. The peptide was counterstained with Alexa Fluor 488-labeled anti-rabbit secondary antibody overnight in PBS containing 5% donkey serum. After unbound dye molecules were washed off with PBS, the sections were incubated overnight with anti-IRAP (D7C5) XP^®^ rabbit monoclonal antibody (Catalog No. 6918, Cell Signaling Technology, MA, United States) labeled with allophycocyanin labeling kit – SH (Dojindo Molecular Technologies, Kumamoto, Japan). The stained samples were dried on glass slides and mounted on cover slips with FluorSave Reagent (Calbiochem, San Diego, CA, United States). Images were acquired on a FV3000 confocal laser scanning microscope (Olympus, Tokyo, Japan) using 488 and 647 nm lasers as excitation light sources.

### Quantification of AVP

The levels of AVP in the brain were measured using an Arg^8^-vasopressin ELISA kit (ab205928, Abcam, Cambridge, United Kingdom) according to the manufacturer’s instructions.

### Cell Culture, Differentiation, and Rhythmic Synchronization

PC12 (pheochromocytoma) cells were cultured in DMEM medium (Nacalai Tesque, Kyoto, Japan) containing heat-inactivated (56°C, 30 min) fetal bovine serum at 37°C in humidified air containing 5% CO_2_. The cells were differentiated with 100 ng/mL of murine NGF-2.5S from Sigma-Aldrich (St. Louis, MO, United States) for 5 days. For synchronization, cells were treated with 50% (v/v) horse serum for 2 h, in accordance with a reported method (Balsalobre et al., 1998), and then kept in the DMEM containing 10% fetal bovine serum medium.

### Western Blot Analysis

A 10-μg aliquot of total protein in the tissue and cell extracts was analyzed by western blotting to detect P-LAP/IRAP (a monoclonal antibody termed GOH-1 was prepared by immunizing mice with recombinant human P-LAP/IRAP), IRAP (D7C5) XP^®^ rabbit monoclonal antibody, IRAP (3E1) mouse monoclonal antibody (Catalog No. 9876, Cell Signaling Technology), and GAPDH (Santa Cruz, CA, United States). Protein blots were probed using an anti-P-LAP antibody followed by an HRP-labeled secondary antibody. The protein bands were detected using an ImageQuant LAS 4000 Mini Luminescent Image Analyzer (GE Healthcare, Chicago, IL, United States) and ECL Prime Western Blotting Detection Kit (GE Healthcare). Densitometric analysis of the antibody response was performed using the ImageJ software.

### Measurement of Cystinyl-Aminopeptidase Activity

Cystinyl-aminopeptidase (CAP) activity was determined using *S*-benzyl-cysteine-4-methylcoumaryl-7-amide (Bzl-Cys-MCA; Bachem, Bubendorf, Switzerland) by modifying a previously described method (Matsumoto et al., 2000). Briefly, 25 μL lysate was mixed with 25 μL PBS containing 200 μM Bzl-Cys-MCA, and incubated at 37°C for 15 min. The concentration of 7-amino-4-methylcoumarin was measured using an SH-9000 Lab multi-microplate reader (Hitachi High Tech, Tokyo, Japan) at excitation and emission wavelengths of 380 and 460 nm, respectively. The measured fluorescence intensities were converted to enzymatic activities using a standard curve generated with 7-amino-4-methylcoumarin.

### Statistical Analysis

All the data reported here are representative of at least 3 independent experiments and are presented as mean ± SE. Statistical analysis was conducted using the Student’s *t*-test and one-way ANOVA; *p* < 0.05 was considered statistically significant. The mean values of each group were compared using the Tukey–Kramer multiple comparison test.

## Results and Discussion

**FIGURE 1:**
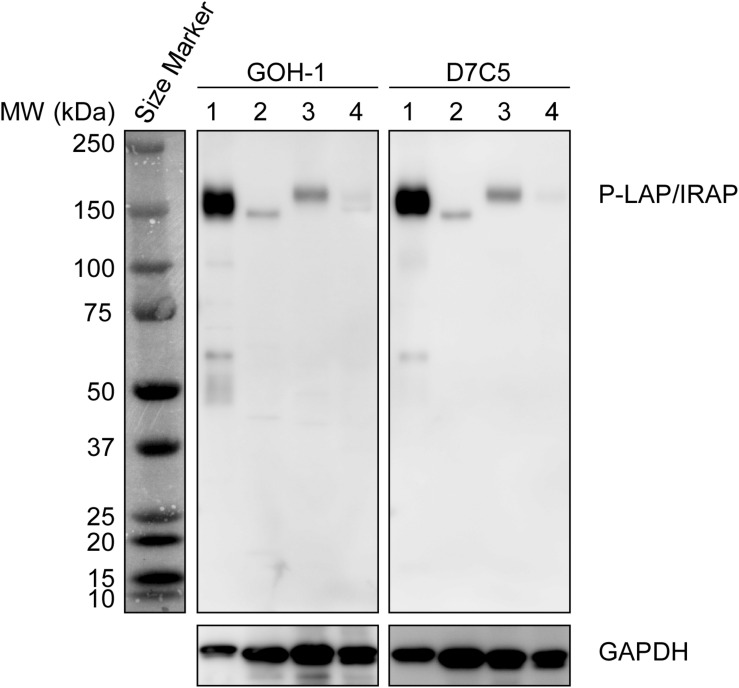
Specific recognition of P-LAP/IRAP by the anti-P-LAP/IRAP antibodies employed in this study. Western blot analyses of PC12 cell lysate and tissue lysates prepared from C56BL/6J mice [detection of P-LAP/IRAP (top) and GAPDH (bottom) as an internal control]. Lane 1: PC12 cells, lane 2: brain, lane 3: heart, and lane 4: liver.

Initially, we compared the specificity of GOH-1 with 2 commercially available anti-P-LAP/IRAP monoclonal antibodies (D7C5 and 3E1) by western blot analysis (Pan et al., 2019; Evnouchidou et al., 2020). As shown in Figure 1, GOH-1, and D7C5 recognized the same protein with a molecular weight of 140–165 kDa, which was expected to be P-LAP/IRAP. As shown previously (Matsumoto et al., 2001), P-LAP/IRAP in the brain has a lower molecular weight than that in the heart, and the liver is associated with minimal expression of the enzyme. On the other hand, 3E1 recognized several unidentified proteins in addition to P-LAP/IRAP. Therefore, we concluded that the GOH-1 monoclonal antibody was suitable for western blot analysis.

### Expression Patterns of P-LAP/IRAP and AVP in Several Murine Brain Sub-regions

Placental leucine aminopeptidase/insulin-regulated aminopeptidase is known to cleave several peptide substrates located in the brain (Matsumoto et al., 2000). Among those tested, vasopressin was the most efficiently cleaved. Furthermore, vasopressin was the first reported physiological substrate of this enzyme (Wallis et al., 2007). Therefore, in this study, we attempted to elucidate the relationship between the expression pattern and levels of P-LAP/IRAP and vasopressin in several sub-regions of the murine brain, with the overall objective of elucidating the roles of P-LAP/IRAP in the brain.

**FIGURE 2:**
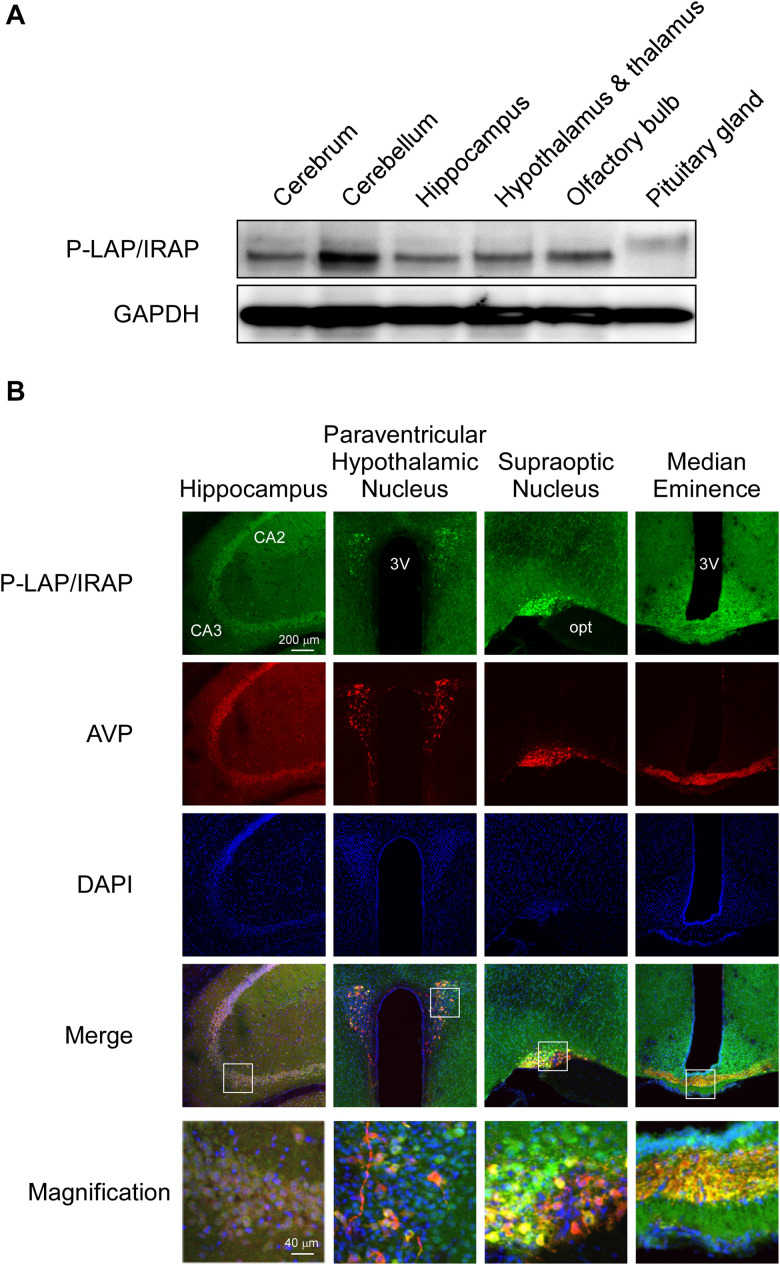
Expression patterns of P-LAP/IRAP and AVP in several sub-regions of the murine brain. **(A)** Western blot analysis of P-LAP/IRAP distribution in several sub-regions of the murine brain. **(B)** Immunohistochemical localization of P-LAP/IRAP (green) and AVP (red) in several sub-regions of the murine brain. Higher-magnification views of the boxed areas in the merged images are shown at the bottom panel. CA2: cornu ammonis 2 area, CA3: cornu ammonis 3 area, 3V; 3rd ventricle, and opt; optic tract.

Figure 2A shows the expression levels of P-LAP/IRAP in several sub-regions of the murine brain. A P-LAP/IRAP immunoreactivity band at a molecular mass of 140 kDa was detected in all the sub-regions except the pituitary gland, which expressed a 165 kDa peptide. We have previously reported the expression of P-LAP/IRAP in several human tissues, including the heart, small intestine, kidney, and brain (Matsumoto et al., 2001). The molecular mass of the enzyme expressed in the brain was slightly lower than that of the enzymes expressed in other tissues. Since the primary sequence of the enzyme contains 18 potential *N*-glycosylation sites (Rogi et al., 1996), differential glycosylation could have led to the observed differences in the molecular mass of the enzyme. Hence, we speculated that, in the murine brain, only the pituitary gland expressed a high-molecular-weight enzyme, presumably because of higher *N*-glycosylation levels. However, we could not exclude the possibility of amino acid deletions from the enzyme expressed in sub-regions of the brain other than the pituitary gland. In fact, we have previously reported 4 amino-acid deletions from the *N*-terminal end of the recombinant human P-LAP/IRAP expressed in CHO cells (Matsumoto et al., 2000).

Next, we performed immunohistochemical analyses using D7C5 to rule out the possibility of non-specific interaction between GOH-1 and sample proteins prepared from several sub-regions of the brain. Frequent usage of D7C5 for immunohistochemical analysis has been previously established (Sun et al., 2014; Evnouchidou et al., 2020).

Figure 2B shows the immunohistochemical localization of P-LAP/IRAP and AVP in various regions of the murine brain.

In the hippocampus, expression patterns of P-LAP/IRAP and AVP exhibited large overlaps. The merged magnified image suggested that a substantial portion of P-LAP/IRAP was co-expressed with AVP, and this co-expression was typically seen in the CA3 to CA2 areas. In the paraventricular hypothalamic nucleus, P-LAP/IRAP was expressed ubiquitously, and strong expression was observed as patches where AVP-producing cells were co-localized with P-LAP/IRAP-expressing ones. A close look at the merged image suggested that higher expression of P-LAP/IRAP in these cells tended to be associated with lower AVP expression and *vice versa*. In the supraoptic nucleus, P-LAP/IRAP- and AVP-expressing cells were also concentrated in the same area. There were two areas showing apparently differential expression patterns, out of which showed both P-LAP/IRAP and AVP expression (as judged by the yellow color in the merged image), and the other predominantly expressed AVP. It was thus plausible that in the former area, P-LAP/IRAP and AVP were co-expressed in the same cells, and in the latter, cells dominantly expressing AVP were localized. P-LAP/IRAP-expressing cells were distributed in a diffused manner throughout the median eminence, whereas the localization of AVP-expressing cells was restricted to neural fibers at its center. Merged images suggested the co-expression of P-LAP/IRAP and AVP in the same cells in the central area. Taken together, although expression patterns of immunostained P-LAP/IRAP and mature AVP showed appreciable overlaps, non-overlapping staining patterns were also observed in several sub-regions of the murine brain. Considering our previous work showing that neuronal cells, but not glial cells, in the brain expresses the enzyme (Matsumoto et al., 2000), it is tempting to speculate that significant portions of neuronal cells co-expressed P-LAP/IRAP and AVP in several sub-regions of the murine brain. In addition, neuronal cells dominantly expressing AVP were also localized adjacent to the co-expressing cells in certain regions.

In the suprachiasmatic nucleus of the hypothalamus that contains a circadian pacemaker, circadian rhythms in vasopressin expression have been reported (Ingram et al., 1998; Mieda et al., 2015). Under LD conditions, AVP levels in the suprachiasmatic nucleus show diurnal rhythmic variations with a peak in the early light phase and a broad trough during the dark phase. Considering that vasopressin is one of the best substrates of P-LAP/IRAP, we compared the expression patterns of the latter with that of vasopressin in the brain hippocampus and pituitary gland. Vasopressin-synthesizing neurons in the paraventricular and supraoptic nuclei in the hypothalamus are known to project to the posterior pituitary (Swaab et al., 1975).

### Diurnal Patterns of P-LAP/IRAP and AVP Expression in the Hippocampus and Pituitary Gland

**FIGURE 3:**
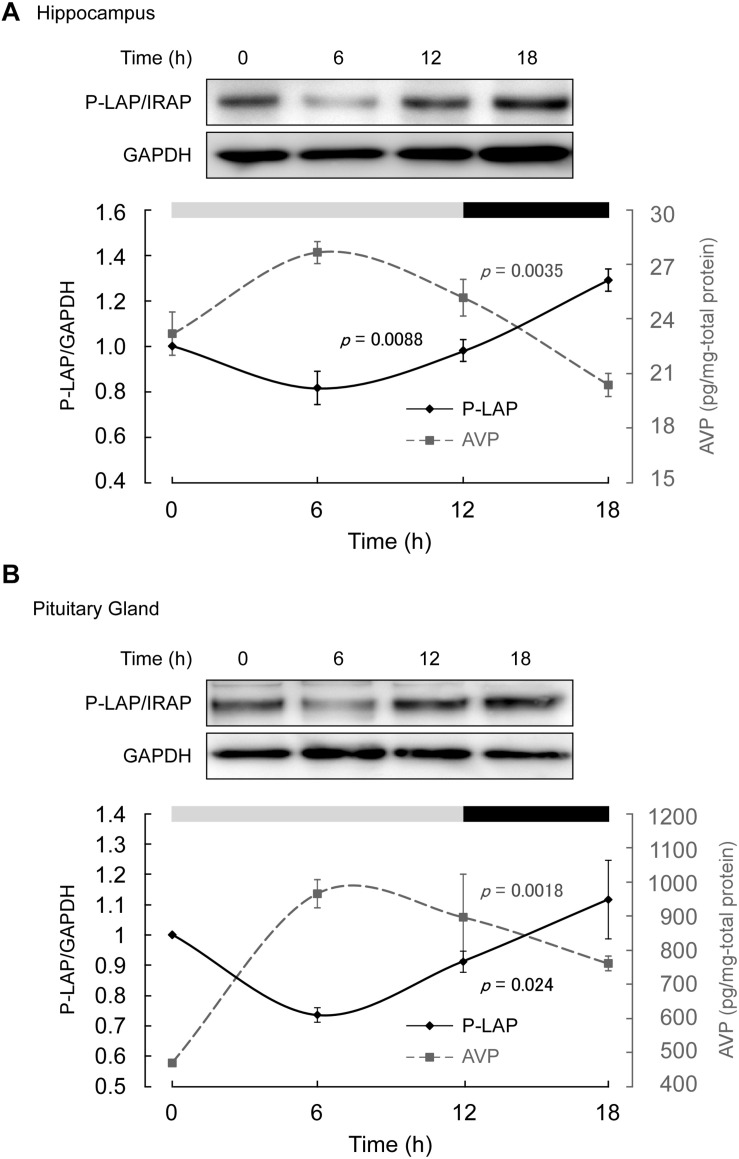
Expression levels of P-LAP/IRAP and AVP in the **(A)** murine hippocampus and **(B)** pituitary gland. The expression level of P-LAP/IRAP was monitored by western blot analysis (top panel) and quantified by densitometric analysis (bottom panel). AVP levels (bottom panel) were monitored using a commercially available ELISA assay kit, as described in section “Materials and methods.” The LD cycle of the sacrificed animals is also shown in the figure.

Mice maintained under controlled environmental conditions with an LD cycle (12 h light, 12 h dark) were employed, as described in section “Materials and methods.” As expected, in the middle of the light phase, higher expression level of AVP was observed in both the hippocampus and pituitary gland. On the other hand, the AVP levels tended to decrease in the dark phase (Figure 3). One-way ANOVA revealed diurnal expression patterns of AVP in both the hippocampus and pituitary gland (*p* = 0.0035 and 0.0018, respectively). These results support the notion that although the experimental period in this study was rather short, diurnal rhythms in vasopressin content in both the hippocampus and pituitary gland could be detected.

On the other hand, P-LAP/IRAP levels in the hippocampus and pituitary gland showed a trend opposite to that of AVP, reaching a minimum value in the middle of the light phase (Figure 3). In the dark phase, an increase in P-LAP/IRAP expression was noted. One-way ANOVA revealed diurnal expression patterns of P-LAP/IRAP levels in both the hippocampus and pituitary (*p* = 0.0088 and 0.024, respectively). Since the P-LAP/IRAP-expressing cells in the hippocampus were located near the AVP-producing ones, it is tempting to speculate that the expression level of P-LAP/IRAP might have affected the level of AVP secreted into the extracellular milieu. When P-LAP/IRAP levels were high, the levels of AVP secreted from the neighboring cells were low, and *vice versa*. Hence, we concluded that the expression of P-LAP/IRAP and AVP showed an antiphasic pattern. In addition, vasopressin mRNA levels in the brain have been reported to be rhythmically dominated by clock genes (Carter and Murphy, 1992; Garbarino-Pico and Green, 2007). To our knowledge, our findings constitute one of the first evidences to suggest that P-LAP/IRAP may contribute to post-translational rhythms in vasopressin expression levels by mediating proteolytic degradation. These results therefore suggest that P-LAP/IRAP modulates the physiological functions of vasopressin in hyperosmolality, memory, learning, and circadian rhythm.

### Circadian Patterns of P-LAP/IRAP in PC12 Cells

In our previous work, we observed that nerve growth factor (NGF) treatment induced the differentiation of rat PC12 cells into neuronal cells and characteristic neurite outgrowth was observed within 2 days (Matsumoto et al., 2001). Additionally, an increase in the expression of P-LAP/IRAP in these cells was observed during differentiation. Considering the diurnal expression pattern of P-LAP/IRAP in the brain, we speculated that the enzyme expression and activity were regulated by the circadian clock in neurons. Therefore, we examined the expression and enzymatic activity patterns of P-LAP/IRAP in differentiated PC12 cells.

**FIGURE 4:**
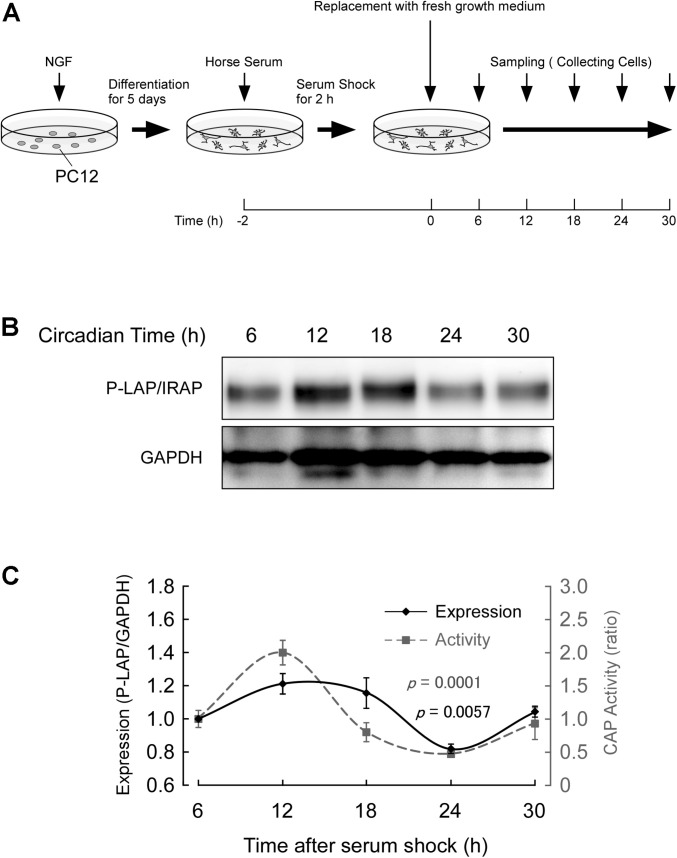
Expression of P-LAP/IRAP in PC12 cells. **(A)** Protocol for P-LAP/IRAP expression in synchronized PC12 cells. NGF-treated PC12 cells were synchronized by the addition of fresh medium containing 50% horse serum. The cells were further incubated for the indicated times and their P-LAP/IRAP expression levels and enzymatic activities were monitored. **(B)** Time-course study (by western blot analysis) of P-LAP/IRAP protein content in PC12 cells after the addition of fresh medium. **(C)** Quantitative analyses of P-LAP/IRAP levels and their enzymatic activity in PC12 cells.

Cells were treated with NGF for 5 days and further incubated in the presence of 50% horse serum for 2 h. After serum shock, these cells were cultured in fresh culture medium (DMEM containing 10% fetal bovine serum) to start the synchronous culture. Then, the cells were collected every 6 h after replacement of the media (Figure 4A) and P-LAP/IRAP expression and CAP activity, which represents P-LAP/IRAP activity, were determined.

Figure 4B shows the changes in immunoreactivity of P-LAP/IRAP in NGF-treated PC12 cells up to 30 h after the serum shock. As expected, an immunoreactivity band at a molecular mass of 140 kDa was clearly detectable. In addition, we repeatedly observed a clear oscillation of the reactivity, with a peak between 12 and 18 h and a trough at 24 h after the serum shock. Figure 4C shows the quantified expression levels of P-LAP/IRAP normalized using the GAPDH level, confirming the changes in the expression level of the enzyme within 30 h after the serum shock. We also monitored CAP activity in these cells and observed an in-phase rhythmic variation of the activity with P-LAP/IRAP expression. These results suggest that the expression of P-LAP/IRAP in NGF-treated neuronal cells shows circadian rhythmicity with synchronized oscillation for at least 30 h after the serum shock. Considering the possible substrates of P-LAP/IRAP in the brain reported so far (Matsumoto et al., 2000; Wallis et al., 2007), this rhythmic pattern in its enzymatic activity may have regulatory roles in neuronal cell processes involving the modulation of the functions of its substrates, such as vasopressin.

In this study, we report possible autonomous expression of P-LAP/IRAP in the hippocampus and pituitary gland of the brain for the first time. Rhythmicity was also observed in NGF-treated PC12 cells, suggesting that it is an intrinsic property of neuronal cells that affects the expression levels of peptide substrates such as vasopressin, somatostatin, and oxytocin in the brain. Reciprocal rhythmic increases and decreases in P-LAP/IRAP and AVP levels in several regions of the brain support the notion that this enzyme regulates peptide levels *via* its enzymatic activity. In addition, it has also been reported that peptide substrates such as vasopressin and oxytocin increase the cell surface expression of P-LAP/IRAP in human umbilical vein endothelial cells and rat kidney cells, respectively, Nakamura et al. (2000), Masuda et al. (2003). It is therefore tempting to postulate a feedback loop between P-LAP/IRAP expression in neuronal cells and AVP secreted from same or neighboring cells. Alternatively, it is also possible that the intrinsic rhythmicity of P-LAP/IRAP expression in these cells solely facilitates its localization in the plasma membrane and thus modulates the level of extracellular AVP produced by these or adjacent cells. More studies are required to elucidate the roles of this rhythmic expression pattern of P-LAP/IRAP in the brain.

## Data Availability Statement

The raw data supporting the conclusions of this article will be made available by the authors, without undue reservation.

## Ethics Statement

The animal study was reviewed and approved by Committee of Teikyo-Heisei university.

## Author Contributions

YG, TN, and MT conceived the study and prepared the manuscript. YG performed all the experiments. KO and AH prepared the monoclonal antibody against P-LAP/IRAP. All authors have read and approved the manuscript.

## Conflict of Interest

The authors declare that the research was conducted in the absence of any commercial or financial relationships that could be construed as a potential conflict of interest.
